# Investigating the Morphogenesis and Replacement of Lamprey Toothlets Using Synchrotron Imaging

**DOI:** 10.1002/jmor.70094

**Published:** 2025-10-21

**Authors:** Madleen Grohganz, Jake Leyhr, Zerina Johanson, Tatjana Haitina, Sophie Sanchez, Kathleen Dollman, Jan Stundl, Marianne E. Bronner, Gareth J. Fraser, Philip C. J. Donoghue

**Affiliations:** ^1^ Bristol Palaeobiology Group, School of Earth Sciences University of Bristol Bristol UK; ^2^ Department of Organismal Biology Uppsala University Uppsala Sweden; ^3^ Department of Biology Duke University Durham NC USA; ^4^ Natural History Museum London UK; ^5^ European Synchrotron Radiation Facility Grenoble France; ^6^ Division of Biology and Biological Engineering California Institute of Technology Pasadena CA USA; ^7^ Faculty of Fisheries and Protection of Waters University of South Bohemia in Ceske Budejovice Vodnany Czech Republic; ^8^ Department of Biology University of Florida Gainesville FL USA

## Abstract

Teeth are a key innovation that underpinned the adaptive radiation of jawed vertebrates; however, their evolutionary origin must lie with the diverse tooth‐like structures of living and fossil jawless vertebrates. Most previous studies have focussed on the extinct stem‐gnathostomes that phylogenetically intercalate the living jawed and jawless vertebrates. The only two extant jawless cyclostome lineages, the lampreys and hagfish bearing keratinous toothlets, have long been overlooked, though they possess complex (but unmineralised) toothlets that some have interpreted as precursors to the teeth of jawed vertebrates. Regardless of whether the toothlets of cyclostomes are homologous or convergent on the teeth of jawed vertebrates, they have the potential to offer unparalleled molecular developmental insights into the evolutionary origin of teeth. To that end, we provide a synthesis of classical literature on cyclostome toothlet structure and development, as a basis for informing future molecular studies, to which we add new insights from X‐ray microtomography of three parasitic lamprey species spanning the breadth of the lamprey crown group. Based on detailed morphological analysis we describe their toothlet replacement mechanism at tissue level and uncover a relationship between toothlet size and the number of replacement cones. All examined species reveal the presence of replacement toothlets, suggesting this replacement mode is a conserved feature of the lamprey crown group. We discuss these results in comparison to hagfish, and conclude that toothlet replacement is a symplesiomorphy of cyclostomes. By describing lamprey toothlet development and replacement and comparing it with gnathostome teeth, this study lays the foundation for research into the development and evolution of teeth and tooth‐like structures across vertebrate lineages.

## Graphical abstract

Toothlet replacement is a conserved feature in lampreys. We describe the mechanism at tissue level and quantify the determining factors, thus providing the basis for studies into a deep homology of cyclostome toothlets and gnathostome teeth.

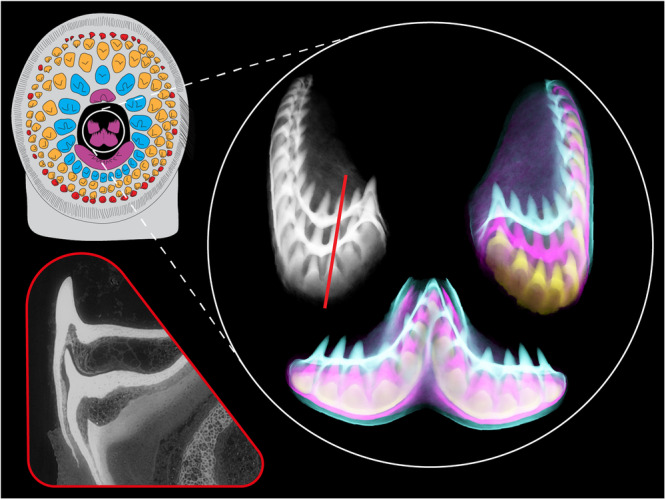

## 1 Introduction

Teeth are a key innovation underpinning the evolutionary and ecological diversification of jawed vertebrates (Gans and Northcutt 1983; Rücklin et al. 2012), as well as a model system for organ development in general (Jernvall and Thesleff 2000; Peters and Balling 1999; Thesleff and Sharpe 1997). Teeth are already present in gnathostomes; they first appear in the extinct placoderm groups Arthrodira (*Compagopiscis*) and Acanthothoraci (*Romundina*) (Rücklin and Donoghue 2015; Rücklin et al. 2012). But the evolutionary relationship of teeth to tooth‐like structures in jawless vertebrates is unclear; to uncover the origin of gnathostome teeth, we need to study the tooth‐like structures of their jawless sister groups to test the homology of these structures to gnathostome mineralised teeth. Relatedly, two hypotheses have been proposed to explain the evolutionary origin of teeth, the ‘inside‐out’ (Johanson and Smith 2005; Smith 2003; Smith and Coates 1998; Smith and Coates 2000; Smith and Coates 2001) and the (revised) ‘outside‐in’ hypotheses (see review in Donoghue and Rücklin 2016; Huysseune et al. 2009; Fraser at al., 2010). The ‘inside‐out’ hypothesis proposes that teeth evolved independently of and before jaws from the specialised tooth‐like structures in the oropharyngeal cavity of jawless vertebrates. According to this hypothesis, teeth have an evolutionary history independent of and preceding the dermal skeleton (Johanson and Smith 2005; Smith 2003; Smith and Coates 1998; Smith and Coates 2000; Smith and Coates 2001). The ‘outside‐in’ hypothesis (Donoghue and Rücklin 2016; Huysseune et al. 2009; Qu et al. 2013) argues that teeth developed in close association with the external odontodes of the dermal skeleton. This is based on the similarity in the structure and development of teeth with dermal denticles, which both develop from a homologous unit termed the odontode (Donoghue 2002; Ørvig 1977; Reif 1982). According to the revised ‘outside‐in’ hypothesis (Huysseune et al. 2009), teeth arose through the spread of odontogenic competence from the external dermis to the oropharynx. This capacity to form internal odontodes was exapted to a tooth function in gnathostomes later in evolutionary history (Grohganz et al. 2024).

To test between these hypotheses, it is pertinent to study the tooth‐like structures of jawless vertebrates from which the teeth of gnathostomes ultimately evolved. So far, much of the debate and hypothesis testing around the evolutionary origin of teeth has focused on the extinct groups of ‘ostracoderms’, phylogenetically intermediate jawless lineages including the groups of osteostracans, galeaspids and heterostracans (Janvier 1996; Smith and Coates 1998). Studying tooth‐like structures associated with the mouth of these fossil stem‐gnathostomes provides an opportunity to test for tooth homology and among the different hypotheses relating to the evolution of teeth. However, this mostly ignores the extant groups of jawless vertebrates and the relationship of their tooth‐like structures to gnathostome teeth. Lampreys, besides hagfish, are the only living jawless sister group of gnathostomes and so they may provide insights into the evolutionary origin of teeth. Hagfishes and lampreys, the cyclostomes, possess toothlets, but unlike teeth in crown‐gnathostomes, they do not consist of dentine (covered with a hard tissue (i.e. enamel or enameloid)), but of keratin. The keratinous dentition of lampreys aids in feeding in their parasitic representatives and the shape and arrangement of these toothlets is commonly used to define lamprey species (Hubbs and Potter 1971; Renaud 2011) and make inferences about their feeding biology (Potter and Hilliard 1987; Renaud et al. 2009). The homology of lamprey toothlets (keratodonts) and the teeth of gnathostomes has been long contested (Dawson 1963; Krejsa et al. 1990a, 1990b; Lethbridge and Potter 1981; Slavkin and Diekwisch 1996; Smith and Coates 1998; Smith and Coates 2000; Smith and Hall 1990; Smith et al. 1996). As a model for experimental study, the living cyclostomes have major advantages over fossil jawless vertebrates in that modern molecular methods for dissecting development can be applied. This is long overdue, allowing us to ask what insights cyclostome toothlets may provide to our understanding of the evolutionary origin of teeth, whether they represent evolutionary precursors of the teeth of gnathostomes, or whether they are convergent, homologous only as ectodermal appendages.

Ectodermal appendages are generally the product of epithelial‐mesenchymal interactions (Chuong 1998; Mikkola 2007; Pispa and Thesleff 2003). The mesenchyme gives the first inductive signal, which is followed by the formation of an epithelial placode that buds in or out of the mesenchyme and subsequently proliferates and differentiates. The epithelium is often ectodermal, but in the case of teeth, it has been shown that the epithelium can be of ectodermal, endodermal or mixed origin (Huysseune et al. 2022; Soukup et al. 2008). The epithelial‐mesenchymal interactions are mediated by a small number of signalling pathways including the Wnt, fibroblast growth factor (Fgf), transforming growth factor β (Tgfβ), hedgehog (Hh) and tumour necrosis factor (Tnf) families, that are widely conserved across species and types of epithelial appendages (Chuong 1998; Pispa and Thesleff 2003). Therefore, we expect the development of all epithelial appendages including keratinous toothlets as well as teeth, to be governed by similar basic mesenchyme‐epithelium signalling pathways. However, there are distinctive differences that set teeth apart from other tooth‐like epithelial appendages, e.g. their growth and development (Grohganz et al. 2024). Teeth are generally characterized by patterns of regeneration and replacement. In jawed vertebrates like sharks this ability to regenerate has been linked to tooth‐specific expression of the stem cell factor sox2, which does not appear e.g. in scales (Martin et al. 2016). Apart from the basic mesenchyme‐epithelium signalling pathways, recent work highlights that the teeth of gnathostomes share highly conserved developmental pathways involving the same set of genes, the so‐called dental gene regulatory network (dGRN) (Fraser et al. 2010; Fraser et al. 2009). No data exists on the genes involved in the development of the keratodonts of cyclostomes, but it could be key to testing the nature of their homology (if any) to gnathostome teeth. However, before it is possible to embark on such a study it is first necessary to understand the classical development of these structures. How exactly do cyclostome toothlets grow?

**Figure 1:**
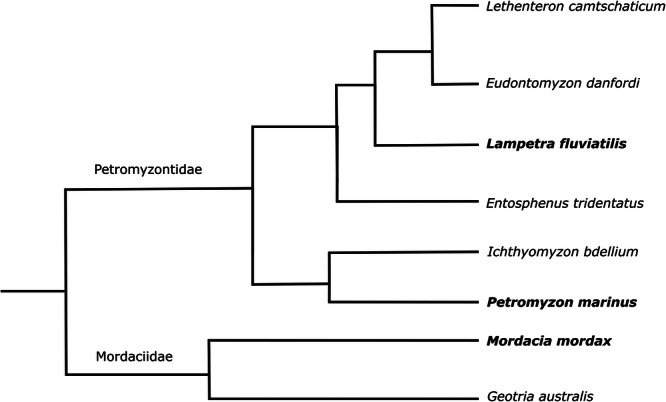
Simplified phylogenetic tree of parasitic lampreys, species examined in this study are indicated in bold. Phylogeny based on Smith et al. (2023) and Brownstein and Near (2023).

Here, we present foundational work into these aspects by characterising cyclostome toothlets, their development and morphogenesis with a specific focus on replacement. First, we review the available literature on these topics, which relied heavily on observations from 2D histological sections and were mostly restricted to *Petromyzon marinus*. Second, we provide new 3D µCT data and expand the study of toothlet morphogenesis to additional lamprey species, *Lampetra fluviatilis* and *Mordacia mordax*. These species hold key positions in the crown group of lampreys (Figure 1). *M. mordax* is a species of the family Mordaciidae which, together with Geotriidae, comprises the Southern Hemisphere lampreys. *P. marinus* and *L. fluviatilis* are species of the Petromyzontidae, the only family in the Northern Hemisphere lampreys (Potter et al. 2015). Together, these species circumscribe the lamprey crown‐group (the clade of living lampreys), allowing us to derive inferences on the nature of the crown lamprey. With this study on the development of lamprey toothlets, we establish a framework on which further molecular genetics studies can be built, investigating a potential deep homology between gnathostome teeth and lamprey toothlets to elucidate the evolutionary origin of teeth.

## 2 Lamprey Oral Hood and Toothlets

### 2.1 General Morphology of the Oral Hood and Toothlets

**Figure 2:**
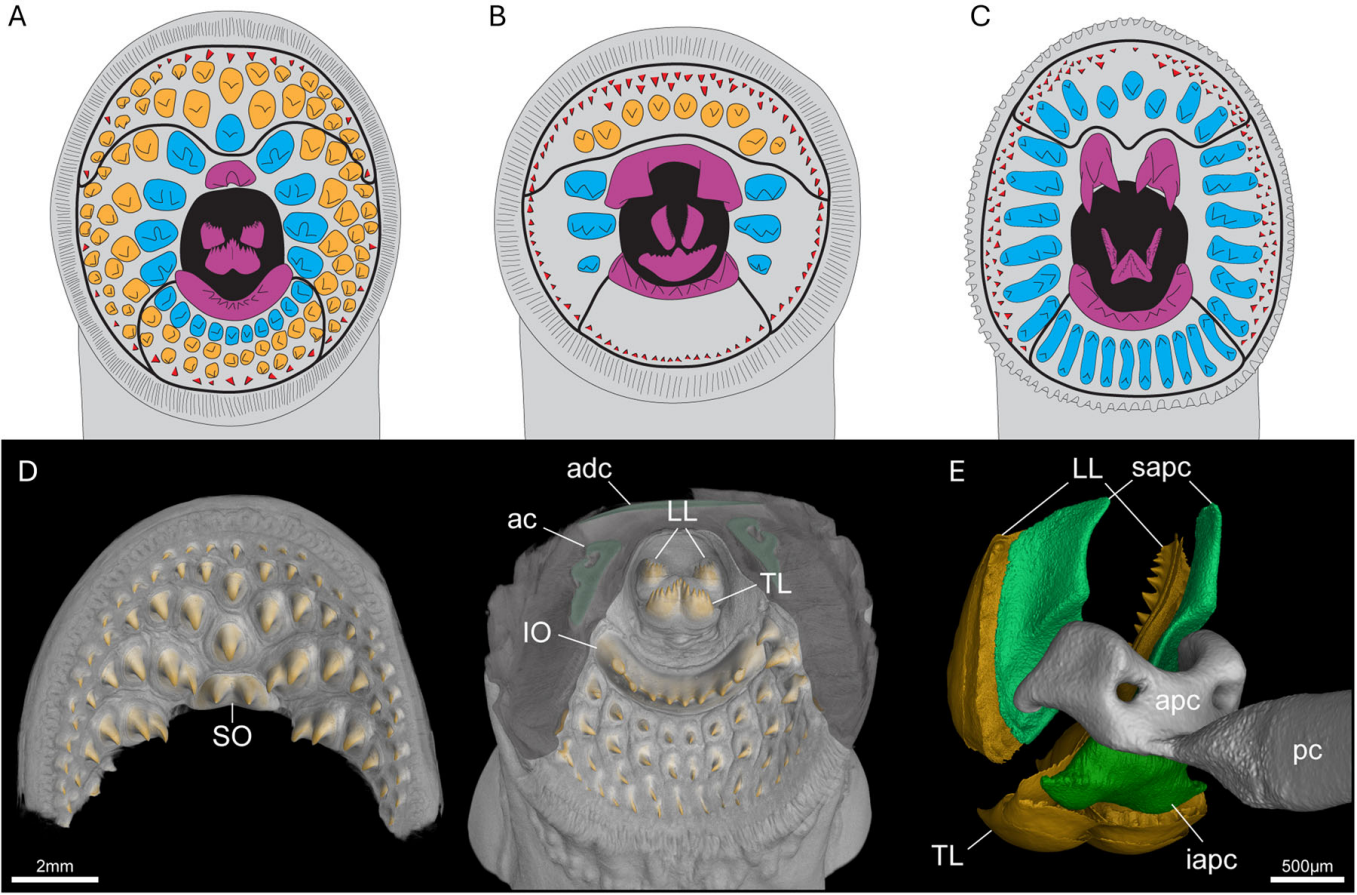
Oral structures of *Petromyzon marinus* (A), *Lampetra fluviatilis* (B), and *Mordacia mordax* (C). Anterior, lateral, and posterior tooth fields are delineated by black lines. Tongue teeth, IO, and SO in purple, and circumoral, intermediate, and marginal hood teeth in blue, orange, and red, respectively. (D) Anteroventral renderings of the anterior and posterior oral hood of a *P. marinus* juvenile, with tooth cusps in orange and cartilage in green; the same colours apply to (E). (E) Posterolateral rendering of the isolated tongue teeth and supporting cartilages. ac = annular cartilage, adc = anterior dorsal cartilage, apc = apical piston cartilage, iapc = infraapical piston mucocartilage, IO = infraoral lamina, LL = longitudinal lingual lamina, pc = piston cartilage, sapc = supraapical piston cartilages, SO = supraoral lamina, TL = transverse lingual lamina.

Hubbs and Potter (1971) introduced a terminology of dentition that we use as a basis to define the different types of toothlets in the oral hood of the three lamprey species we examine (see Figure 2):

a. Tongue teeth: sit in the centre of the oral hood and are bilaterally symmetrical. They consist of a transverse lingual lamina (TL) and a pair of longitudinal lingual laminae (LL) bearing multicuspid teeth.
b. Hood teeth: arranged into posterior field (PF), lateral field (LF) and anterior field (AF), described relative to the esophageal opening in the centre of the hood. Generally, the hood teeth are largest towards the esophageal opening and decrease in size towards the edge of the oral hood.
  – Circumoral teeth (in blue): row of teeth forming a circle directly around the esophageal opening excluding the infraoral and supraoral teeth.
  – Intermediate teeth (in orange): between the circumorals and marginals.
  – Marginal teeth (in red): small teeth at the margin of the oral hood.
c. Infraoral tooth (IO): a wide, curved midline multicuspid tooth that sits directly below the esophageal opening, also referred to as the infraoral lamina.
d. Supraoral tooth (SO): teeth that sit directly above the esophageal opening, also referred to as the supraoral lamina.

The arrangement and morphology of these toothlets varies between species and is related to feeding modes, e.g. blood feeding versus flesh feeding (Figure 2B, C; Renaud et al. 2009). For example, the form of the SO is highly divergent between *P. marinus*, *L. fluviatilis*, and *M. mordax* (Figure 2A‐C). In *P. marinus*, the bicuspid SO is a similar size and shape to the other big teeth in the LF, while in *L. fluviatilis* it is so large as to span the width of the esophageal opening in a similar manner to the IO. In place of a single bicuspid SO, *M. mordax* displays bilateral SOs, each with three elongated cusps. Other notable differences between the species include the reduced number of teeth in the hood of *L. fluviatilis*, particularly in the PF (Figure 2B).

### 2.2 Description of the Piston Cartilage in *Petromyzon Marinus*

The piston cartilage is closely associated with the oral hood of scavenging lampreys and is a key skeletal component in generating the force necessary to feed (De Beer 1937; Johnels 1948). It is also found in fossils, indicating that the piston cartilage dates back to early in the evolutionary history of lampreys (Chang et al. 2006; Gess et al. 2006; Janvier 2007).

Detailed descriptions of the structure of the tongue teeth in relation to the piston cartilage are generally lacking in the literature and so we segmented out the relevant structures from the oral hood of a *P. marinus* juvenile specimen (Figure 2E). Viewed posterolaterally, the bi‐lobed shape of the apical piston cartilage is clear, supporting the anterodorsal supraapical piston cartilages. It is upon these supraapical cartilages that the pair of longitudinal lingual lamina are mounted anteriorly. Ventral to the longitudinal lingual lamina is the transverse lingual lamina, supported by a mass of soft mucocartilage that we term the infraapical piston mucocartilage (iapc), distinct from the previously mentioned hard cartilages (see S1).

### 2.3 Lamprey Replacement Toothlets and Their Epithelial Layers

Previous studies indicate the presence of replacement toothlets forming underneath the functioning cone/horn cap potentially satisfying the tooth criterion of replacement from a ‘generative set’ (see Reif 1982). The first record of multiple horn caps appears in Beard (1888, p. 499), who describes “three horny cusps […] lying one upon the other, and each arising in a special groove at the base of the tooth” in *P. marinus*. However, Beard did not interpret them as replacement structures; instead, he interpreted them as merely strengthening the outermost cone. Warren (1902) also observed the presence of several stacked cones in histological sections through the head of *P. marinus*. He was the first to interpret them as successional teeth developing beneath the functional tooth and also confirmed that the cone of ‘odontoblasts’ is purely epidermal.

**Figure 3:**
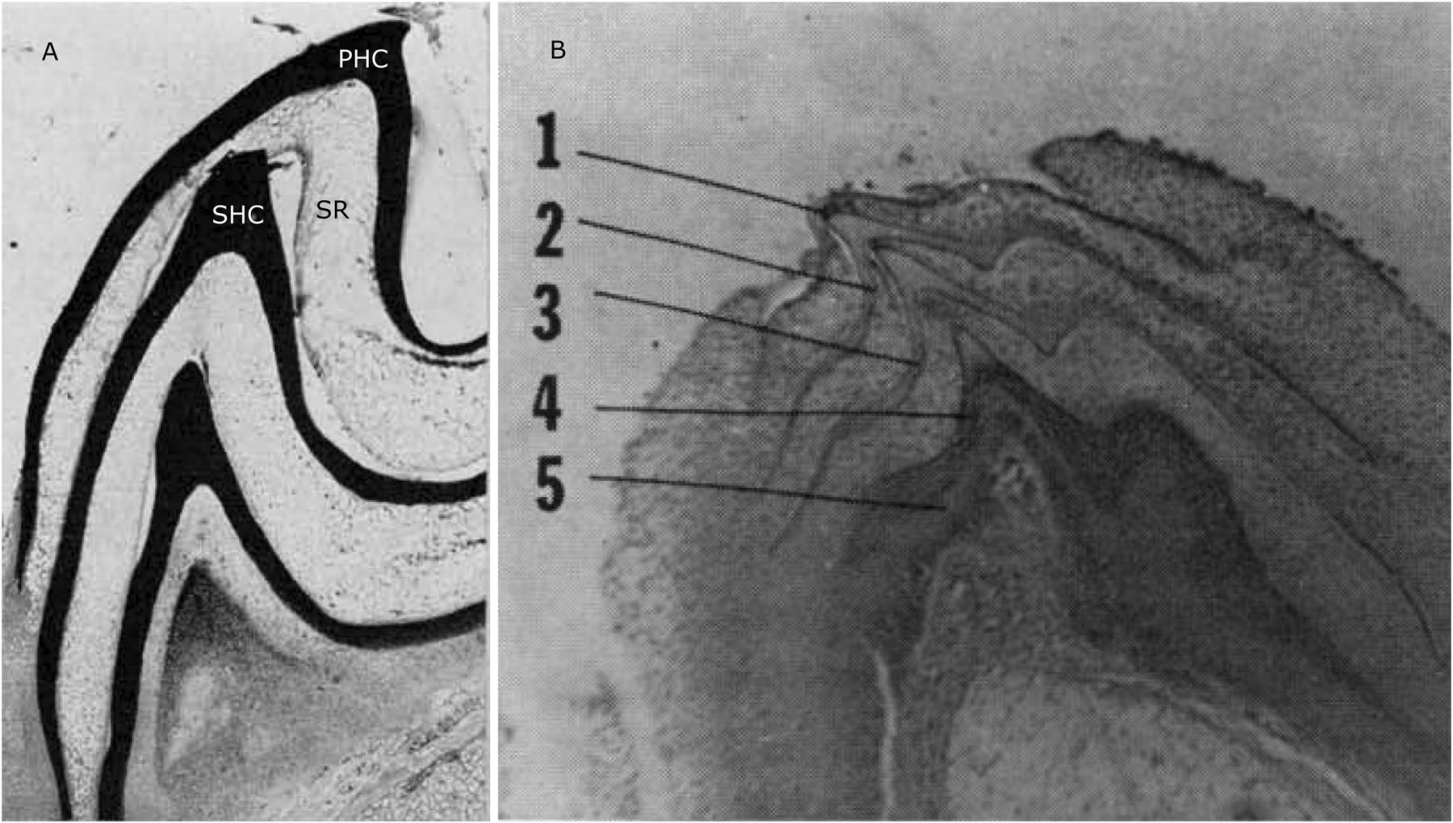
Sections through toothlets of *P. marinus* showing replacement toothlets. (A) section through a toothlet of *P. marinus* with horn caps stained dark, degenerating stellate reticulum (SR) between primary horn cap (PHC) and secondary horn cap (SHC), adapted from Sognnaes and Lustig 1955. (B) Section through tongue tooth in a late stage metamorphosis *P. marinus* with one primary and four replacement horn caps, adapted from Manion and Piavis 1977.

**Figure 4:**
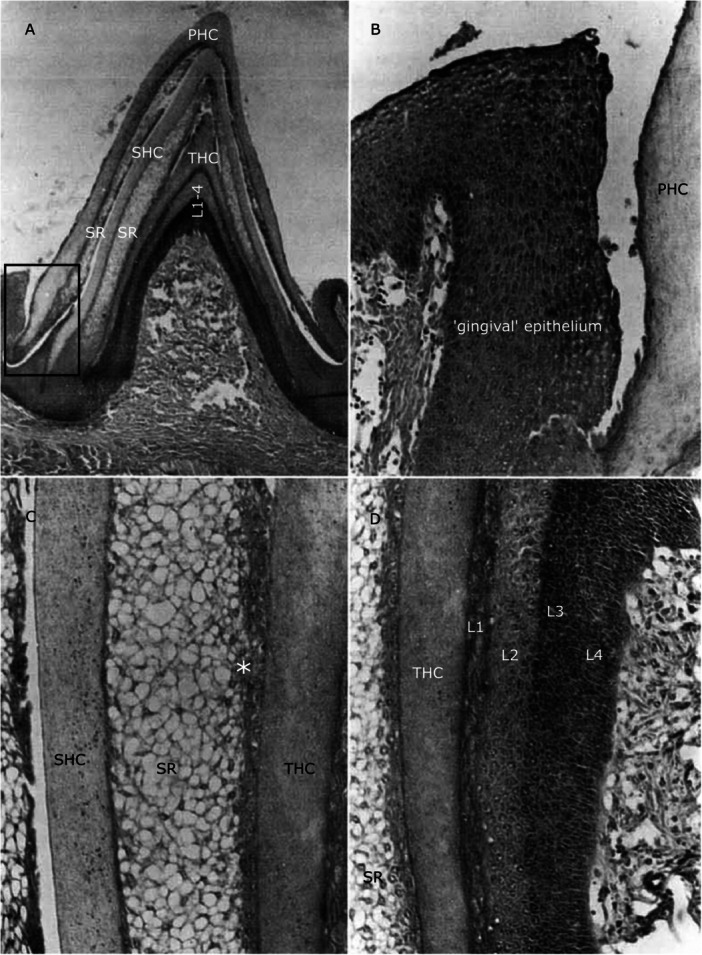
Sections through toothlets of *P. marinus* illustrating the different epithelial layers. (A) Section through a fully developed toothlet of *P. marinus* with three keratinous cones stacked on top of each other (primary horn cap (PHC), secondary horn cap (SHC) and tertiary horn cap (THC)), rectangle indicates position of close‐up in (B). (B) Close‐up of the ‘gingival’ epithelium next to the primary horn cap (with a space between the two). (C) Close‐up of stellate reticulum (SR) underlying the secondary horn cap (SHC), asterisk marks flattened epithelial cells towards the tertiary horn cap. (D) Close‐up of epithelial layers 1‐4 (L1‐L4) underlying the tertiary horn cap (THC). Adapted from Trott and Lucow 1964.

**Figure 5:**
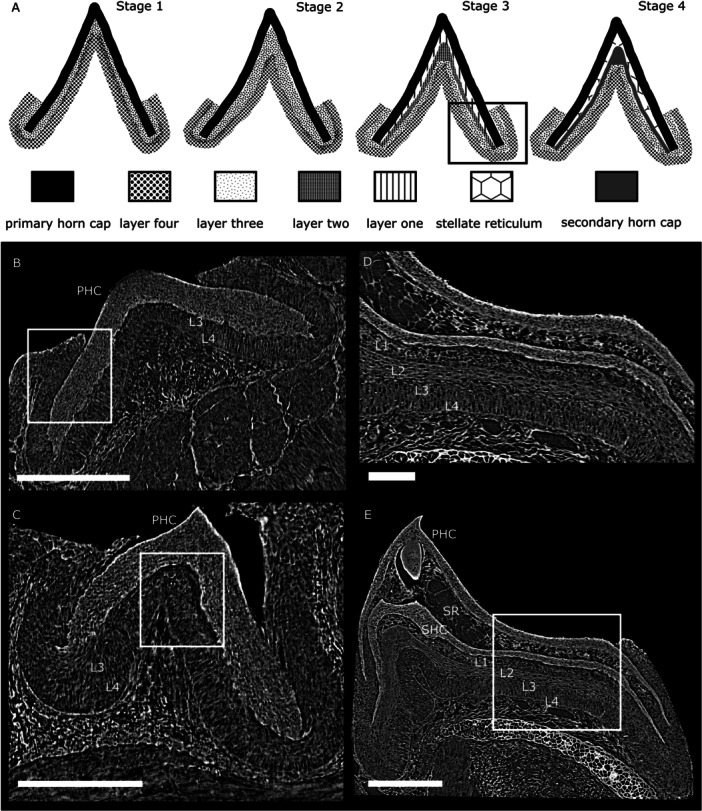
Reconstruction of replacement process at tissue level and supporting µCT tomography data of *P. marinus* illustrating the different stages and epithelial tissues: (A) Schematic drawings of different stages and epithelial tissues involved in replacement toothlet development, rectangle in Stage 3 corresponds to location of section in (D). (B) Section through a toothlet in Stage 1 with primary horn cap (PHC) and underlying epithelial layer 3 (L3) and layer 4 (L4), rectangle indicates close association between ‘gingival’ epithelium and the primary horn cap. (C) Section through a toothlet in Stage 2 with thickened epithelial layer 3 (L3) and layer 4 (L4) underlying the primary horn cap (PHC), rectangle indicates the beginning differentiation of L3 and L4 into layer 1 and layer 2. (D) Section through a toothlet with completely developed epithelial layers layer 1 (L1), layer 2 (L2), layer 3 (L3) and layer 4 (L4) underlying the horn cap. (E) Section through a toothlet beyond stage 4 showing a differentiated epithelium ready to form a second replacement cone with layer 1 (L1), layer 2 (L2), layer 3 (L3) and layer 4 (L4) underlying the secondary horn cap (SHC) and stellate reticulum (SR) underneath the primary horn cap (PHC), rectangle indicates position of close‐up in (D). Scale bars represent: 50 μm (B), 200 μm (C), 60 μm (D), 150 μm (E).

Sognnaes and Lustig (1955) carried out histochemical studies on histological sections of *P. marinus* toothlets using different staining methods and described the epithelial layers for the first time. They found the outer/primary horn cap to be the most keratinised part of the lamprey toothlet, the degree of keratinisation decreasing towards the younger, inner replacement cones. The primary horn cap is underlain by a layer of loose, stellate reticulum (Figure 3A). This is formed by prickle cells, keratin‐producing epidermal cells, and their lowest layer appears flattened towards the secondary/replacement horn cap (see also Figure 4C). Sognnaes and Lustig (1955) argue that continuous use of the outermost toothlet leads to degeneration of the reticulum, loss of the primary horn cap and emergence of the secondary one. At the base they found a cone‐shaped connective‐tissue papilla. In our µCT tomography data of *P. marinus* we can confirm the presence of the tissue layers described by Sognnaes and Lustig (1955). We also observe a primary horn cap underlain by stellate reticulum on top of a secondary horn cap (see Figure 5E).

**Figure 6:**
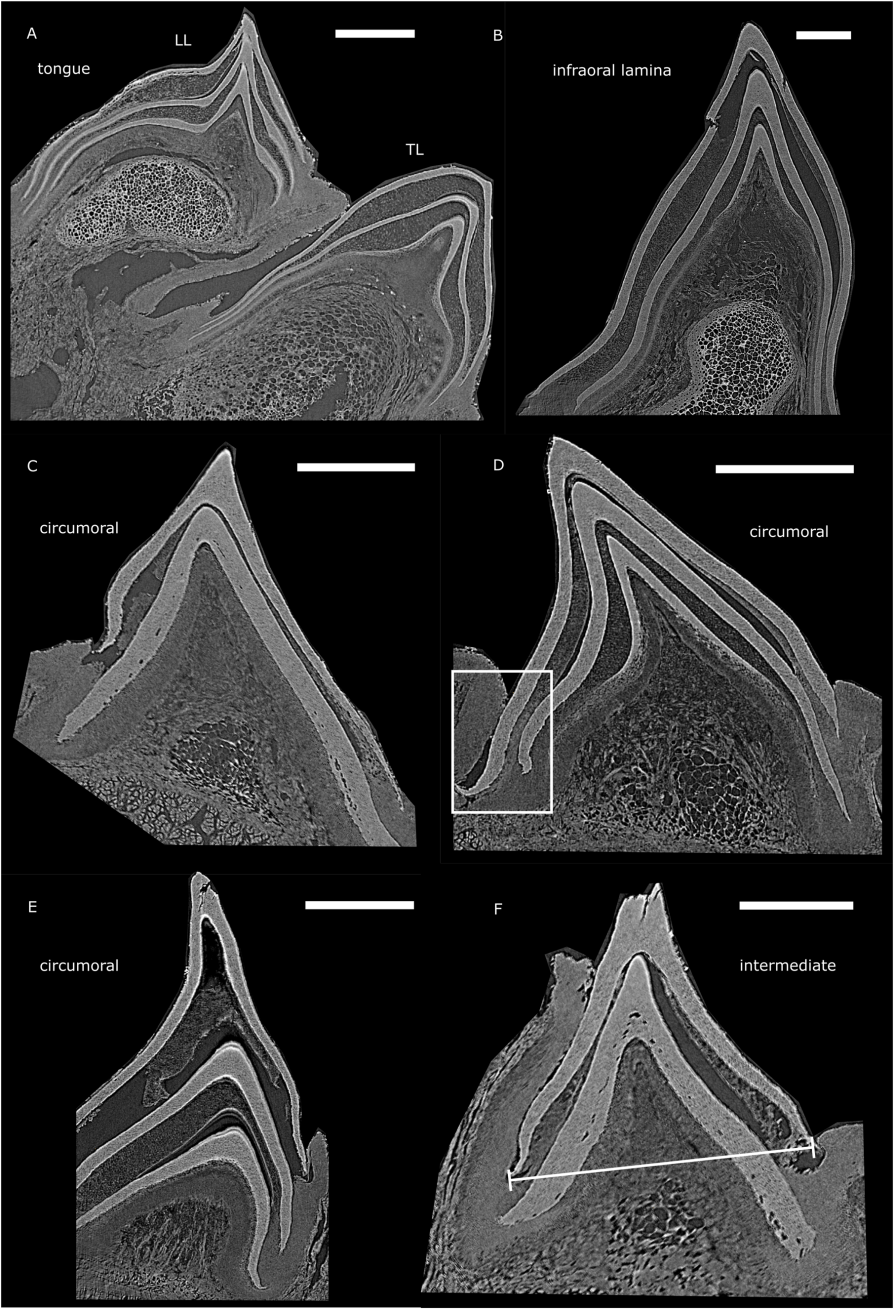
srXTM tomography data from *P. marinus* adult toothlets: (A) Section through the tongue teeth of *Petromyzon marinus*, left longitudinal lingual lamina (LL) and right transversal lamina (TL) with two replacement cones respectively. (B) Section through the infraoral lamina with two replacement cones. (C—E) Sections through circumoral hood teeth in the lateral field with one replacement cone (C) and two replacement cones (D, E), rectangle in (D) indicates space between the ‘gingival’ epithelium and the primary horn cap. (F) Section through an intermediate hood tooth in the posterior field with one replacement cone, white line represents location of toothlet diameter measurement. Scale bars represent 500 μm.

Trott and Lucow (1964) investigated the oral hood of *P. marinus* and found that the circularly arranged toothlets increase in size from the periphery of the oral hood towards the oesophagus. We make the same observation not only for *P. marinus*, but also for *L. fluviatilis* and *M. mordax* (see above, Figure 2A‐D). The size of the teeth decreases from the circumoral teeth next to the esophageal opening towards the intermediate teeth and the marginals at the oral hood periphery. Trott and Lucow (1964) also describe a fold of epithelium at the margin of the outermost cone with a space towards the toothlets (Figure 4A, rectangle and 4B), which they propose to be analogous to the gingiva in mammals. We confirm the presence of this epithelial rolling in our *P. marinus* specimens and observe its origin from the bottom‐most layer of epithelium underlying the innermost cone. In toothlets with replacement cones the space observed by Trott and Lucow (1964) is present (Figure 6D, rectangle). In specimens without replacement cones the ‘gingival’ epithelium is also present but appears in close association (without a space) with the toothlet (see Figure 5B, rectangle). We argue that the space between the ‘gingival’ epithelium and the outermost cone probably develops in a later stage, when the toothlets have grown underlying replacement cones and are approaching the loss of the primary horn cap.

Underlying the replacement cone, Trott and Lucow (1964) describe four tissue epithelial layers (Figure 4D): the first layer (L1) adjacent to the cone consists of flattened epithelial cells, the second layer (L2) of large oval cells, the third layer (L3) consists of large oval prickle cells gradually moving into the fourth layer (L4) of more elongated cells. There is no clear distinction between the third (L3) and the fourth layer (L4). We also observe these four layers in our *P. marinus* specimens (Figure 5). Trott and Lucow (1964) also propose that the fully developed tooth consists of three keratinous cones stacked on top of each other and arising from the oral epithelium at their base (see Figure 4A).

### 2.4 The Replacement Mechanism of Lamprey Toothlets

Previous authors were not able to derive a mechanistic explanation of lamprey toothlet replacement at a tissue level. Our tomography data indicate that in toothlets with only one functioning cone, this cone is underlain directly by a layer of epithelium that consists of tissue layer 3 (L3) and layer 4 (L4) (Figure 5A, Stage 1; Figure 5B). In preparation for the development of the first replacement cone, this epithelium thickens (Figure 5A, Stage 2; Figure 5C) and differentiates into the four epithelial layers described by Trott and Lucow (1964) (layer 1‐4 (L1‐L4); Figure 5A, Stage 3; Figure 5D). Subsequently layer 2 (L2), that consists of large oval cells, keratinises and gives rise to the first replacement cone (Figure 5A, Stage 4). As soon as the keratinisation of layer 2 (L2) starts, the overlying layer 1 (L1) (consisting of flattened epithelial cells) starts to degenerate into the stellate reticulum observed by Sognnaes and Lustig (1955) (Figure 5A, Stage 4; Figure 5E, SR). We propose that the degeneration into stellate reticulum is not primarily controlled by use of the primary horn cap but correlates with the development of a replacement horn cap. Sognnaes and Lustig (1955) also describe a layer of flattened cells right on top of the secondary horn cap (see Figure 4C, asterisk). We interpret this as the remnants of layer 1 (L1) (originally consisting of flattened cells), which degrades into stellate reticulum during replacement cone formation. Underlying the newly formed secondary replacement cone are epithelial layer 3 (L3) and layer 4 (L4), from which a new replacement cone can now form following the process described above (Stage 2‐4).

### 2.5 Determining Factors of Replacement Cone Formation

Manion and Piavis (1977) were the first to study the dentition of *P. marinus* across different life stages and investigated the factors determining the number of replacement cones. They observed a varying number of replacement cones depending on the type of tooth (tongue or hood), the developmental stage (juvenile or adult), and physiological state of the animal (e.g. post‐spawning). They found as many as one primary and four replacement horn caps in a late‐metamorphosis lamprey before the onset of feeding (Figure 3B). The largest number of replacement teeth was present in the tongue ‐ the primary feeding instrument potentially subject to the most rapid replacement. Senescent animals lacked replacement cones and generally had fewer toothlets probably due to physiological deterioration after spawning. They concluded that *P. marinus* systematically replaces its teeth (instead of loss by attrition). This process involves upward displacement by the substitute horn cap until the primary cone separates and is pushed away by the secondary horn cap.

We build on the study of Manion and Piavis (1977) and investigate the number of replacement cones based on statistical analysis and the factors determining replacement in various lamprey species spanning the whole crown group of lampreys.

### 2.6 Replacement in Adults of *Petromyzon marinus*

The examined tongue teeth of the adult *P. marinus* specimens show two sets of replacement toothlets in the transverse lingual lamina as well as the paired longitudinal lingual laminae (Figure 6A).

**Figure 7:**
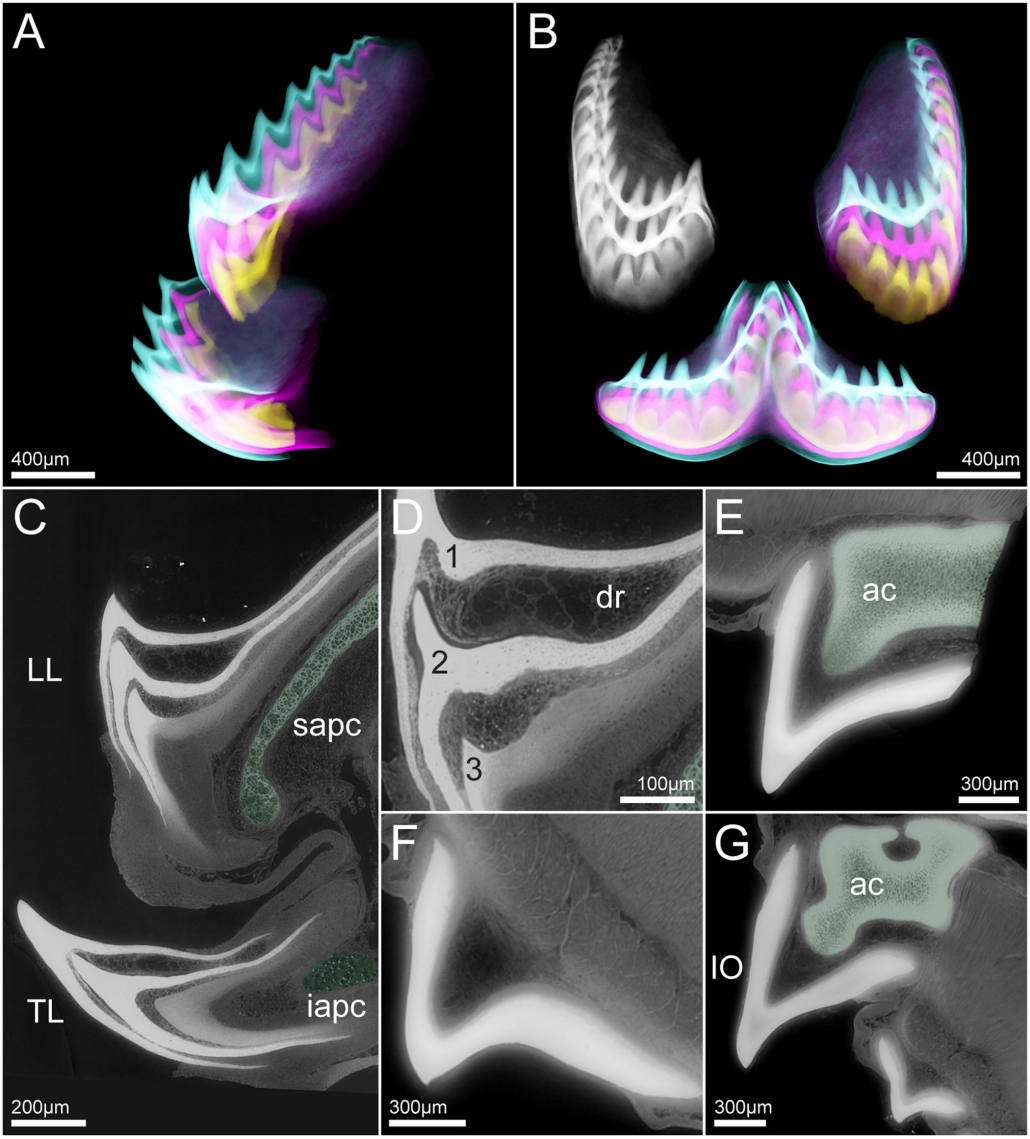
DICE‐PPC‐SRμCT tomography data from *P. marinus* juvenile toothlets. Lateral (A) and anterior (B) X‐ray renderings of the tongue teeth. (C) Section through the tongue teeth. (D) Zoom in on the three stacked cones of the longitudinal lingual lamina (LL). (E) Section through the supraoral lamina (SO). (F) Section through an intermediate hood tooth from the lateral field. (G) Section through the infraoral lamina (IO) and a circumoral hood tooth from the posterior field. LL = Longitudinal lingual lamina, TL = Transverse lingual lamina, IO = Infraoral lamina, ac = annular cartilage, dr = degenerated reticulum, iapc = infraapical piston mucocartilage, sapc = supraapical piston cartilage.

In the infraoral lamina the individual cusps have a mean diameter of about 2000 μm (n = 2) and show two replacement cones underneath the functioning one (Figure 6B). Toothlet cap diameter measurements are based on virtual sections through the synchrotron tomography data. The toothlets have a long asymmetrical axis (see Figure 7C) and perpendicularly to this, a short symmetrical axis (see Figure 6A). The short axis was used for the cap diameter measurements. A cutting plane was determined through the cusp of the toothlet and perpendicular to its base to capture the maximum extent of the toothlet's short axis. Toothlet diameter measurements were taken at the short axis of the outermost cone at its base (see line in Figure 6F).

The circumorals in *P. marinus* have a broad base, they occur as single‐cuspid teeth in the anterior and posterior field and are fused into sets of two cusps in the lateral field (see Figure 2A). The individual cusps of the circumorals have a mean diameter of about 1700 μm (*n* = 5) and show replacement structures varying in number from one to two (Figure 6C‐E). The intermediate teeth of *P. marinus*, sampled from the anterior, lateral and posterior field, are generally single‐cuspid, often with a more slender base than the circumorals and have a mean diameter of about 1200 μm (*n* = 6) with one replacement structure underneath the functioning cone (Figure 6F).

### 2.7 Replacement in Juveniles of *Petromyzon marinus*

The examined tongue teeth of the juvenile *P. marinus* specimens show two sets of replacement toothlets in the transverse lingual lamina as well as the paired longitudinal lingual laminae (Figure 7A‐D).

In the infraoral lamina the individual cusps have a mean diameter of about 1000 μm (n = 8), the individual cusps of the supraoral lamina have a diameter of around 1100 μm (n = 1). In both the infraorals and supraorals we find no evidence for replacement (Figure 7E and G).

In the *P. marinus* juveniles, the division into circumorals, intermediates and marginals is the same as in the adult specimens (see Figure 2A). However, the absolute size of the teeth is generally smaller in the juvenile; the mean cusp diameter is about 600 μm (*n* = 22) for the circumorals, 600 μm (*n* = 44) for the intermediate teeth and 300 μm (*n* = 21) for the marginals. None of these show any signs of replacement in any field (anterior, posterior or lateral) (Figure 7F‐G).

### 2.8 Replacement in Adults of *Lampetra fluviatilis*

**Figure 8:**
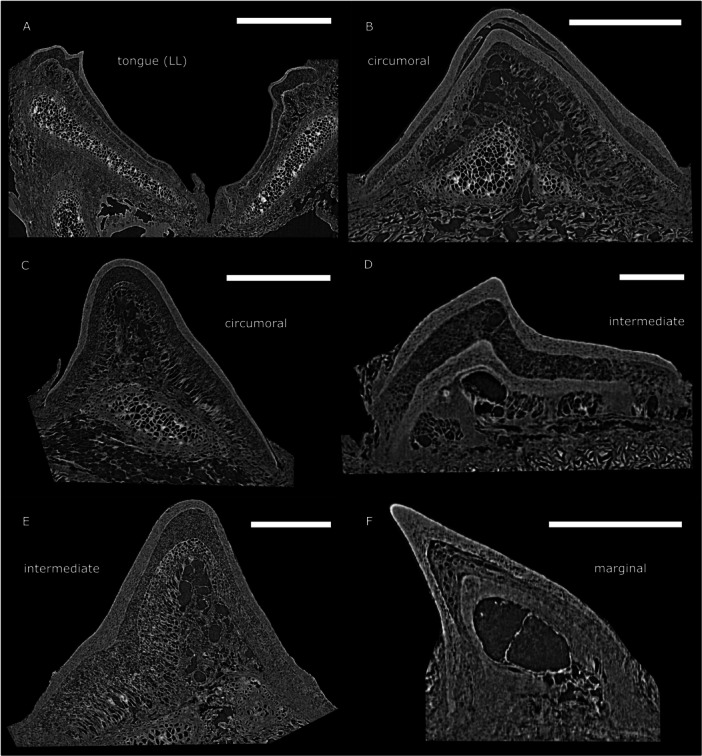
Toothlets of *L. fluviatilis* adults: (A) Section through the longitudinal lingual lamina (LL) tongue tooth with one replacement cone. (B–C) Sections through circumorals in the lateral field with one replacement cone (B) and no replacement cone (C). (D–E) Sections through intermediate hood teeth in the anterior field with one replacement cone (D) and no replacement cone (E). (F) Section through a marginal tooth in the anterior field without replacement. Scale bars represent: 1000 μm (A), 500 μm (B‐D), 250 μm (E, F).

The examined tongue teeth of the adult *L. fluviatilis* specimens show one replacement structure underneath the functioning cone in the tongue tooth (Figure 8A).

The circumorals in *L. fluviatilis* can only be found in the lateral field (see Figure 2B). They have a broad base and occur fused into sets of two or three cusps. The individual cusps of the circumorals have a mean diameter of about 900 μm (*n* = 10) and show replacement structures varying in number from one in the two‐cuspid teeth (Figure 8B) to none in the three‐cuspid teeth (Figure 8C). The intermediate teeth of *L. fluviatilis* can only be sampled from the anterior field. They are generally single‐cuspid, often with a more slender base than the circumorals and have a mean diameter of about 900 μm (*n* = 4) with one replacement structure (Figure 8D) or none replacement structure (Figure 8E) underneath the functioning cone. The marginals lining the outer edge of the oral hood are even smaller in size with a mean diameter of about 200 μm (*n* = 6) and no replacement structures visible (Figure 8F).

### 2.9 Number of Replacement Cones Is Controlled by Toothlet Diameter

We carried out statistical analyses (general linear model and step model selection) on the measurements from the *P. marinus* and *L. fluviatilis* specimens to disentangle the factors influencing toothlet diameter (see S2 for toothlet measurements and R code).

**Figure 9:**
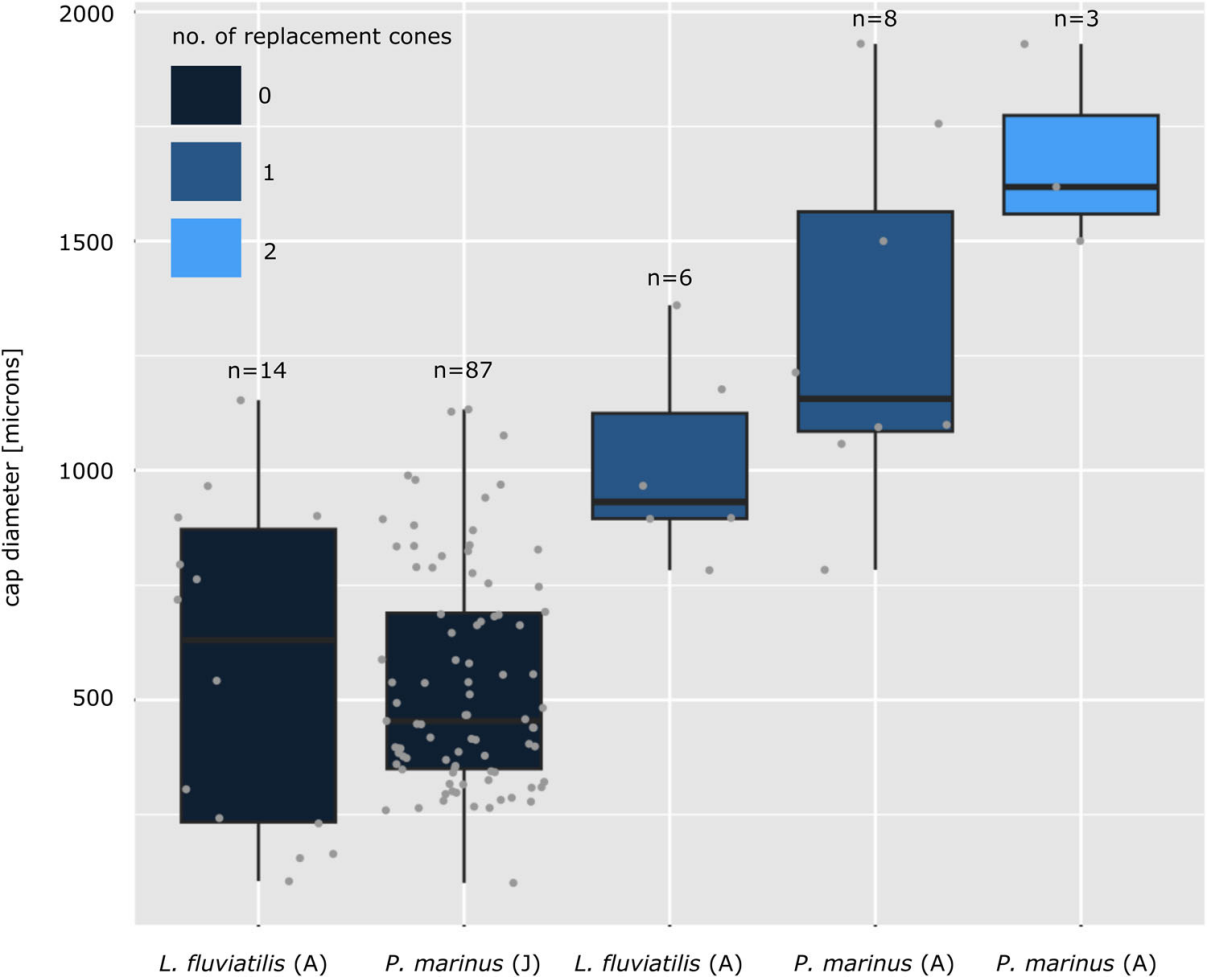
Relationship of lamprey toothlet cap diameter and number of replacement cones. Boxplots of toothlet cap diameter measurements of *L. fluviatilis* and *P. marinus* adult (A) and juvenile (J) specimens sorted by number of replacement cones.

Our model indicates that cap diameter is best described by the factors of life stage, species, number of replacement cones, tooth type (circumoral, intermediate, marginal) and number of cusps (single‐, bi‐ or three‐cuspid); the field location (AF, PF, LF) does not play a major role. The first three factors (bearing the most weight) are displayed in a boxplot diagram (Figure 9). The median toothlet diameter is about 600 μm for *L. fluviatilis* adults without replacement cones (*n* = 14), 500 μm for *P. marinus* juveniles without replacement cones (*n* = 87), 900 μm for *L. fluviatilis* adults with one replacement cone (*n* = 6), 1200 μm for *P. marinus* adults with one replacement cone (*n* = 8) and 1600 μm for *P. marinus* adults with two replacement cones (*n* = 3). Wilcoxon tests of the individual groups without replacement cones show no significant difference in toothlet diameter between the *L. fluviatilis* and *P. marinus* groups (*p*‐value = 0.9569). The same is true when comparing the individual groups with one replacement cone; no significant difference in toothlet diameter can be detected between *L. fluviatilis* and *P. marinus* (*p*‐value = 0.1419). However, there is a significant difference in toothlet diameter between groups with no replacement cone and one replacement cone irrespective of species (see bold p‐values in Table 1). The difference between the groups with no replacement cone and two replacement cones is significant for both *L. fluviatilis* (*p*‐value = 0.002941) and for *P. marinus* (*p*‐value = 0.003477), whereas the difference between the groups with one replacement cone and two replacement cones is only significant for *L. fluviatilis* (*p*‐value = 0.02381), but not for *P. marinus* (*p*‐value = 0.1826).

**Table 1:** p‐values of Wilcoxon tests comparing cap diameters of different lamprey groups shown in boxplots.

	*P. marinus* juvenile 0 replacement	*L. fluviatilis* adult 1 replacement	*P. marinus* adult 1 replacement	*P. marinus* adult 2 replacements
*L. fluviatilis* adult 0 replacement	0.9569	0.0259*	0.0004191***	0.002941**
*P. marinus* juvenile 0 replacement	X	0.0005022***	1.546e‐05****	0.003477**
*L. fluviatilis* adult 1 replacement	X	X	0.1419	0.02381*
*P. marinus* adult1 replacement	X	X	X	0.1826

Our data show that in the tongue of all specimens, independent of species and life stage, at least one (and up to two) replacement toothlets are present. This is consistent with findings from the previous literature described above. The tongue is the only tooth type that already shows replacement cones in the juvenile life stage as the main organ used for feeding straight after metamorphosis. In the hood, the development of replacement cones seems to be controlled by toothlet diameter. The results of our statistical analyses indicate that groups with no replacement cones show significantly different toothlet diameters to groups with replacement cones (Figure 9). The development of the first replacement cone is initiated when the toothlet reaches a size of about 900–1200 μm. In toothlets below this size we normally do not observe any replacement structures in the oral hood. Generally, juvenile specimens do not reach this size of toothlets in the hood due to their smaller body size and therefore do not show replacement in their hood teeth. In the adults the first toothlets to reach this size and develop replacements are the circumorals (plus infraorals and supraorals) followed by the intermediates (the marginals do not reach the replacement size limit even in the adults). This sequence also seems to reflect the importance of the different tooth types for feeding and their position with regard to the esophageal opening. The tongue sits right at the centre of the esophageal opening and is the main organ for feeding and therefore prioritised regarding replacement (already in the juveniles). In the adults replacement follows in the circumorals (plus infraorals and supraorals), which sit close to the esophageal opening and later the intermediates positioned closer to the edge of the oral hood. The toothlets sitting close to the esophageal opening (circumorals) play a more crucial role in feeding (grasping and rasping) than the more marginal toothlets (intermediates and marginals). The circumorals reach a bigger size and start to replace earlier in ontogeny.

### 2.10 Replacement in Adults of the Short‐Headed Lamprey (*Mordacia mordax)*

**Figure 10:**
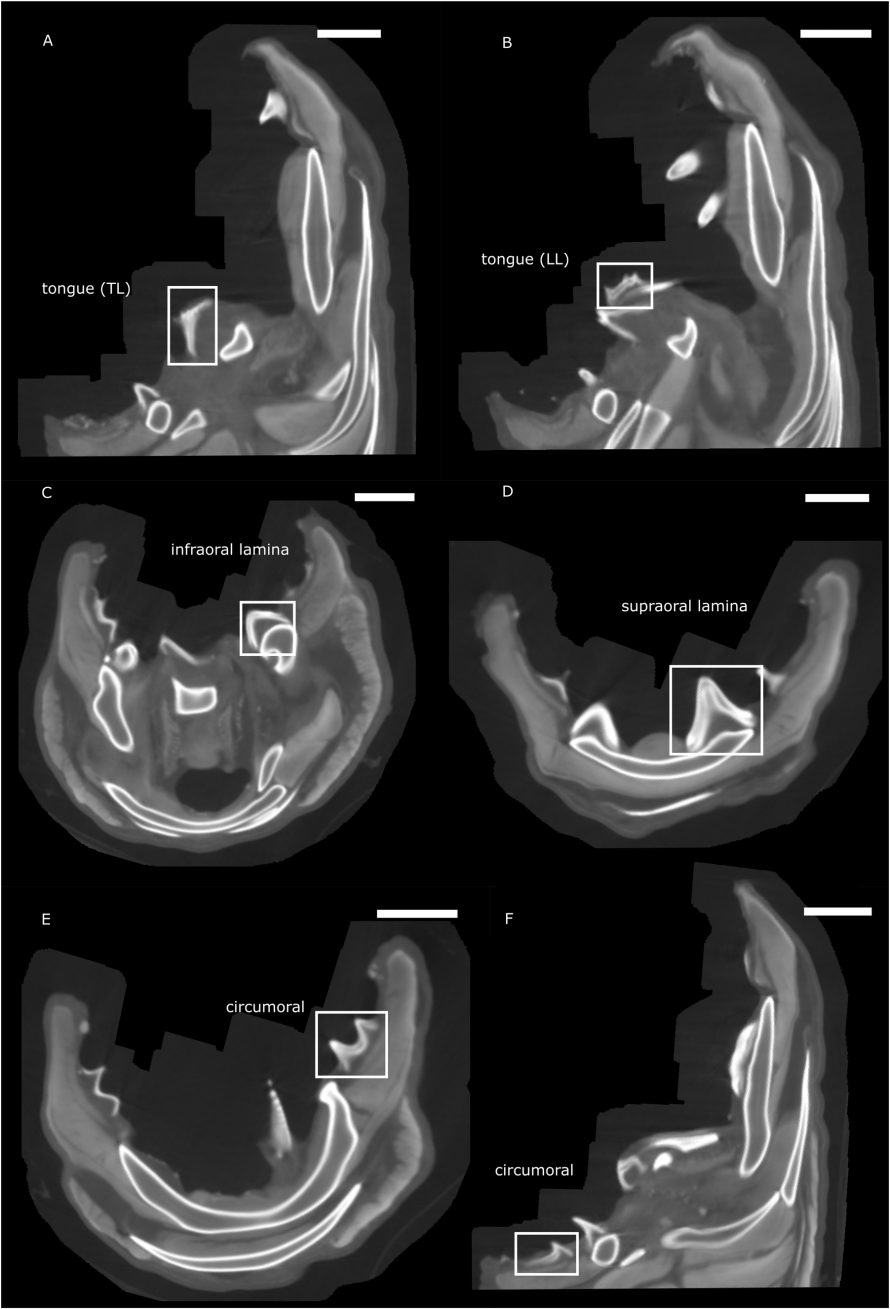
micro‐CT tomography data of *M. mordax* adult toothlets. (A, B) Sections through the tongue teeth*:* (A) Transverse lamina (TL) with one replacement cone. (B) Longitudinal lingual lamina (LL) with one replacement cone. (C) Section through the infraoral lamina with one replacement cone. (D) Section through a three‐cuspid tooth of the supraoral lamina with one replacement cone. (E) Section through a two‐cuspid circumoral tooth in the lateral field with one replacement cone. (F) Section through a single‐cuspid circumoral tooth in the posterior field with one replacement cone. Rectangles mark the respective tooth. Scale bars represent: 3000 μm (A–C, E), 1000 μm (D), 2000 μm (F).

We also investigated X‐Ray tomography data characterising the oral hood of *M. mordax*. However, the lower resolution of these data in comparison to the previously‐described synchrotron tomography data complicates the identification of replacement structures. Nevertheless, in the tongue teeth we find a replacement cone in the transverse lingual lamina as well as the paired longitudinal lingual laminae (Figure 10A‐B). The curved infraoral lamina is located directly below the tongue; its individual cusps are about 800–1200 μm in diameter and show a replacement structure (Figure 10C). The supraoral lamina consists of two big tricuspid toothlets that sit right at the upper part of the esophageal opening (see Figure 2C). Its individual cusps are about 1300‐1700 μm in diameter and show a replacement structure underlying the functioning tooth (Figure 10D). The circumorals appear as individual cusps with broad bases in the anterior field (see Figure 2C), with cusp diameter of about 1000–1200 μm. In the lateral and posterior field the circumorals radiate from the esophageal opening in the form of bi‐cuspid (lateral field) and single‐cuspid (posterior field) toothlets with broad bases with individual cusp diameter of about 1000–1200 μm (lateral field) and around 800 μm (posterior field). The circumorals show a replacement cone (Figure 10E‐F). Their broad bases extend towards the margin of the oral hood (see Figure 2C); intermediate hood teeth or marginals can not be identified in this tomography data.

### 2.11 Replacement as a Conserved Feature in Lampreys

We observe replacement toothlets in all examined species of northern lampreys (*P. marinus, L. fluviatilis*) and southern lampreys (*M. mordax*). This indicates that the formation of replacement toothlets is a conserved feature across the lamprey crown group. A recent study (Wu et al. 2023) described two new species of stem‐lamprey from the Middle‐Late Jurassic Yanliao Biota of Northern China. Their feeding apparatus is well‐preserved with a well‐toothed oral disc in the anterior and lateral fields that resembles the southern hemisphere lamprey *Geotria australis*. Synchrotron tomographic characterization of the oral discs of these fossil specimens would provide a test for the presence of replacement structures and whether this is also a conserved feature of the lamprey total‐group.

### 2.12 Comparison With Toothlets in Hagfish

**Figure 11:**
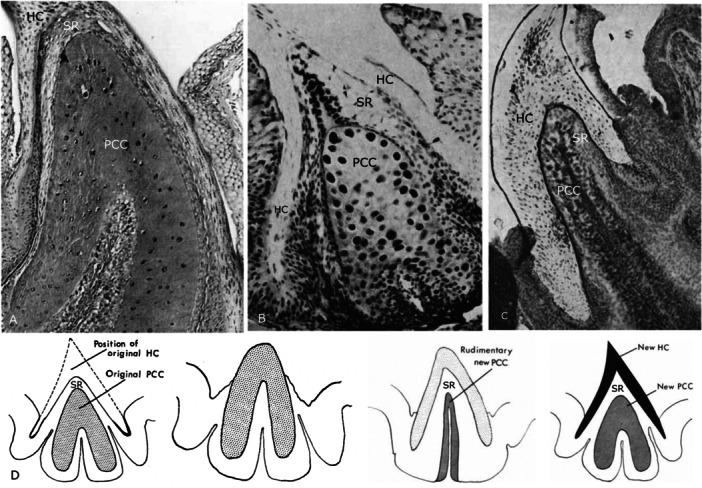
Sections through hagfish toothlets illustrating the different epithelial layers and schematic drawings of toothlet replacement process. (A, B) Sections through fully developed toothlets showing the horn cap (HC) separated from the pokal cell cone (PCC) by stellate reticulum (SR): (A) *Polistotema stoutii*, adapted from Trott and Lucow 1964. (B) *Myxine glutinosa*, adapted from Dawson 1969. (C) Section through a newly formed toothlet of *Myxine glutinosa* with horn cap (HC) and a thin pokal cell cone (PCC) separated by stellate reticulum (SR), adapted from Dawson 1969. (D) Schematic drawings of toothlet replacement process in hagfish, adapted from Dawson 1969.

Crown group lampreys show a mode of replacement with replacement cones forming underneath the functioning horn cap before use. The other group of living jawless fish, the hagfish, have also been described as replacing their toothlets. However, little is currently known about tooth replacement in the group of hagfish. Hagfish are generally described as opportunistic scavengers (Auster and Barber 2006; Martini et al. 1998), but *Neomyxine* has also been observed to feed predatory on free‐swimming prey (Zintzen et al. 2011). Contrary to lampreys, hagfish use their whole body to resist the pressure of their tooth plates during feeding and form a manipulating body knot as leverage (Clark and Summers 2012; Uyeno and Clark 2015). The teeth of hagfish are keratinous and consist of an outer cone‐shaped horn cap (HC), beneath which lies a layer of stellate tissue (intermediate epithelium) and the pokal cell cone (PCC) (epithelial tissue), from which the replacement cone develops (Figure 11A‐C) (Dawson 1969). This structure strongly resembles the lamprey toothlets consisting of an outer primary horn cap (PHC), the stellate reticulum (SR) and the underlying epithelial layers, from which the replacement cone develops (see above). When the HC is lost in hagfish (experimentally or naturally), the intermediate epithelium (stellate tissue) degenerates as the PCC keratinises and converts into a new functioning HC (see schematic drawings in Figure 11D). The original pulp epithelium differentiates into intermediate epithelium (stellate tissue) and PCC to prefabricate a new set of toothlet precursors (the complete process taking about 90 days) (Dawson 1969) (see also Figure 11C). In lampreys a very similar replacement process is observed including the obliteration of the stellate reticulum as new functioning toothlets grow and keratinise underneath to replace the outer cone (see Figure 5A Stage 4; Figure 5E). We also observe the recurring differentiation of the basal epithelial layers to form new underlying replacement cones (see Figure 5E). Lamprey replacement cones are identical to the primary horn cap, they develop as a set of one or more fully keratinised replacement cones underneath the functioning cone. In hagfish the degree of keratinisation differs with the PCC only fully keratinising after the HC is lost (see Figure 11D). Also, in hagfish tooth replacement of all teeth seems to occur synchronously at regular intervals, which is supported by the fact that the PCCs of all tooth rows are in the same stage of development and that the naturally shedding animals detach all tooth rows at the same time (Dawson 1969).

Lampreys, on the other hand, show toothlets in varying stages of development with various numbers of replacement cones, they replace their toothlets asynchronously with prefabricated fully keratinised cones already underlying the outermost cone (see our results above). Despite these differences, both lampreys and hagfish show profound similarities in the structure of their keratodonts and the way they replace them, indicating that the mode of replacement observed in lampreys is a conserved feature of the cyclostome group.

### 2.13 Comparison of Cyclostome Toothlets With Gnathostome Teeth

Teeth can be defined based on various criteria. The topology criterion requires that teeth are located in the oral cavity and develop from oral epithelium; they do not necessarily need to be associated with the jaws, as developmental independence of these two modules has been shown (Schilling et al. 1996). Regardless, there is no expectation that teeth and jaws evolved in concert and, indeed, it has been argued that teeth evolved before jaws (Smith and Coates 1998; Smith and Coates 2000; Smith and Coates 2001). The structure/composition criterion of homology implies that teeth show an odontode structure (shared with the dermal skeleton (Keating et al. 2015)) consisting of dentine, that might be capped with hard tissue (enamel/enameloid) and fixed by bone of attachment (Patterson 1977). The developmental criterion entails that teeth are replaced via loss (Reif 1982) or successional apposition (Rücklin et al. 2012; Rücklin et al. 2021; Smith 1985) and develop as discrete morphogenetic modules that lack evidence of continuous growth between adjacent odontodes.

**Figure 12:**
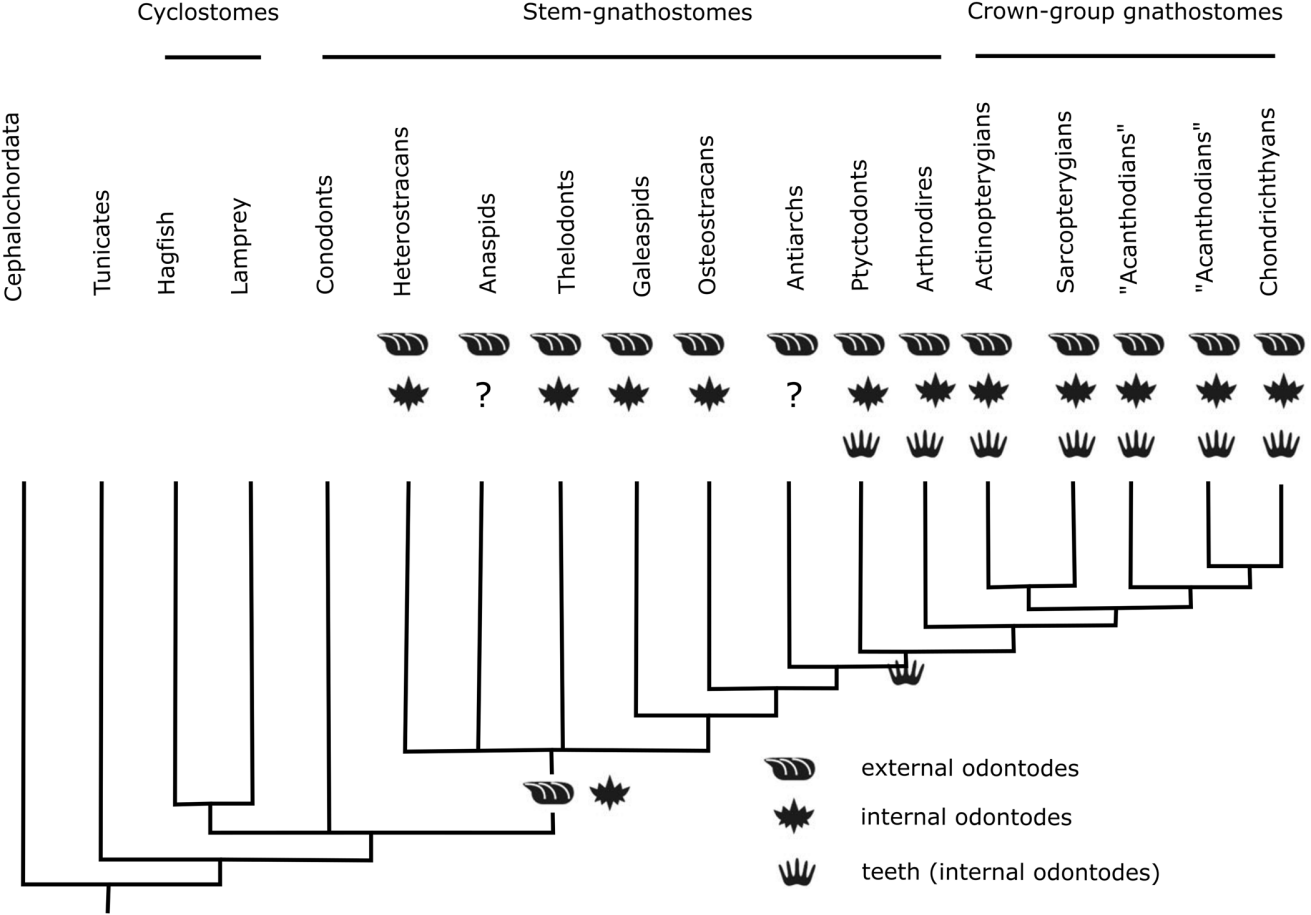
Distribution of external odontodes, internal odontodes and teeth (as a subcategory of internal odontodes), plotted on a phylogenetic tree of vertebrates. Phylogeny adapted from Johanson et al. 2019. Question marks denote ambiguous records, empty tips are not applicable.

Lamprey toothlets satisfy the topology criterion, as they develop from oral epithelium inside the oral cavity. They also meet the developmental criterion, as they show replacement of toothlets via loss of the primary horn cap and the emergence of a ‘prefabricated’ replacement cone (Reif 1982). However, cyclostome toothlets fail on the structure/composition criterion. They do not present the typical odontode structure of internal/external odontodes; consisting of keratin instead of dentine with an enamel(oid) cap. Previously, enamel protein antigens were reported from the teeth of Pacific hagfish (*Eptatretus stoutii*) (Slavkin et al. 1983), suggesting the conservation of enamel throughout vertebrate evolution. But this has later been refuted by studies on brown hagfish (*Paramyxine atami*), which found neither enamelin nor amelogenin in the toothlets (Yokoyama and Ishiyama 1998). Venkatesh et al. (2014) also confirmed with genomic analyses that enamel matrix proteins are not present in cyclostomes. However, many aspects of cyclostome biology are extremely derived and unrepresentative of the ancestral cyclostome or vertebrate condition (Donoghue 2017; Heimberg et al. 2010) and so it remains possible or even likely that their toothlets are an unmineralised vestige of mineralized counterparts in their antecedents. Support for this view may be found in the proposal of homology between the mineralized elements of the extinct jawless conodonts and keratinous toothlets of cyclostomes (Krejsa et al. 1990a, 1990b). The detail of this hypothesis has been rejected because, unlike the replacement toothlets of cyclostomes, the toothlets of conodonts underwent episodic growth (Aldridge and Donoghue 1998; Donoghue and Aldridge 2001; Donoghue 1998; Donoghue and Purnell 1999), but this rejection is also based in part on the hypothesis that conodonts are stem‐gnathostomes (Donoghue et al. 2000) and more recent phylogenies have instead resolved conodonts as stem‐cyclostomes (Miyashita 2020; Miyashita et al. 2019; Miyashita et al. 2021; Reeves et al. 2023; Terrill et al. 2018). As such, conodonts may reflect the ancestral condition for cyclostome toothlets, not least since the paraconodont forebears of euconodonts appear to be paraphyletic (Murdock et al. 2013; Sweet and Donoghue 2001). The question then remains as to whether cyclostome and gnathostome oral odontodes are homologous. Murdock et al. (2013) rejected homology between conodont elements and gnathostome teeth on the basis that their obvious similarities (Donoghue 1998) were acquired in euconodonts from simpler, less extensively mineralised paraconodonts, though even they exhibit odontode‐like character (Dong et al. 2005; Murdock et al. 2013). The most obvious objection to homology is the lack of phylogenetic continuity between these structures, a challenge that has confronted all attempts to identify teeth‐precursors among jawless stem‐gnathostomes (Donoghue and Rücklin 2016; Donoghue and Smith 2001; Rücklin et al. 2011), though this does not preclude the hypothesis that oral odontodes were exapted recurrently to tooth functions among stem and crown‐gnathostomes alike (Grohganz et al. 2024). Indeed, almost all stem‐gnathostomes, comprising the so‐called ‘ostracoderms’ (heterostracans, anaspids, thelodonts and osteostracans) and the placoderms, share an extensive dermal armor of external odontodes as a conserved feature (Figure 12) and various of these groups possess internal odontodes (Figure 12). Testing hypotheses of homology among these structures may be impossible for the extinct groups, though it remains possible to test the ‘inside and out’ hypothesis of deep molecular homology rooted in a common gene regulatory network of all odontodes (Fraser et al. 2010; Fraser et al. 2009) by applying modern molecular methods to characterizing the development and replacement of lamprey toothlets for comparison to their better characterised counterparts, the teeth and scales of crown gnathostomes. Comparing the gene regulatory networks active in gnathostome teeth (Fraser et al. 2009) and lamprey toothlets will help to understand whether they represent evolutionary precursors of, or a parallel evolution to, the teeth of gnathostomes. In either instance, they will provide insights into the evolution of this model organogenic system.

## 3 Conclusion

We provided a review of the literature on lamprey toothlets, their morphogenesis and replacement (mostly restricted to the model species *P. marinus*). Our new µCT tomography data support the findings of these previous studies on lamprey replacement toothlets. We also expand these observations to additional parasitic lamprey species showing that toothlet replacement is a conserved feature in the lamprey crown group. Additionally, our high‐resolution synchrotron tomography data enables us to describe the toothlet replacement mechanism in detail at tissue level and quantify the factors determining replacement. This mechanism of replacement via loss is absent from the placoderms, the most basal jawed vertebrates. This study lays the foundation for future investigations into the genetic regulation of lamprey toothlets and their relationship with gnathostome teeth. We provide the developmental basis for studies into a deep homology of the toothlets in jawless vertebrates and the teeth of gnathostomes, which have the potential to further our understanding of the evolutionary origin of teeth.

## 4 Materials and Methods

The *Lampetra fluviatilis* specimens were obtained from a commercial supplier for fishing bait (Baitbox). The *Petromyzon marinus* specimens were obtained from the USGS, euthanised, and fixed in 4% PFA. The specimens were then gradually dehydrated in 100% ethanol before being dissected.

Specimens dehydrated at the University of Bristol (UK) were then critically point dried at the Wolfson Bioimaging Facility (University of Bristol, UK) and characterized tomographically at the X02DA TOMCAT beamline of the Swiss Light Source, Paul Scherrer Institute in Villigen, Switzerland. Synchrotron Radiation X‐ray Tomographic Microscopy (srXTM; Donoghue et al. 2006) was performed with voxel dimensions of 1.625 μm and 0.65 μm. Reconstructed slice data derived from the tomography scans were analysed and manipulated using Avizo software for computed tomography at the University of Bristol.

The juvenile *Petromyzon marinus* specimen processed at Uppsala University (Sweden) was stained for 1 day in a solution of 1% iodine in 100% ethanol and subsequently scanned at BM05 of the European Synchrotron Radiation Facility ‐ Extremely Brilliant Source (ESRF‐EBS) in Grenoble, France. Propagation phase‐contrast synchrotron radiation microcomputed tomography (DICE‐PPC‐SRμCT) was performed as previously described by Leyhr et al. (2023) with voxel sizes of 3 μm and 0.727 μm. Reconstructed jpeg2000 image stacks were imported into VGStudio MAX (v2023.3) for manual segmentation and rendering. All the husbandry and experiments were in accordance with California Institute of Technology Institutional Animal Care and Use Committee protocol #IA23‐1436.

The data of *Mordacia mordax* specimen YPM:VZ:YPM ICH 006162 (Vertebrate Zoology Division ‐ Ichthyology, Yale Peabody Museum) was accessed on MorphoSouce (www.morphosource.org): ark:/87602/m4/573778 (diceCT scan of whole specimen, voxel size of 42.521 μm). Yale University provided access to the data, the collection of which was funded by oVert TCN; NSF DBI‐1701714; NSF DBI‐1701769.

## Author Contributions

**Madleen Grohganz:** Investigation, formal analysis, visualisation, writing—original draft, writing – review and editing. **Jake Leyhr:** Investigation, formal analysis, visualisation, writing – review and editing. **Zerina Johanson:** Conceptualisation, formal analysis, supervision, writing – review and editing. **Tatjana Haitina:** Resources, writing – review and editing, **Sophie Sanchez:** Resources, writing – review and editing. **Kathleen Dollman:** Methodology, writing – review and editing. **Jan Stundl:** Resources. **Marianne E. Bronner:** Resources. **Gareth J. Fraser:** Conceptualisation, supervision, writing – review and editing. **Philip C. J. Donoghue:** Conceptualisation, methodology, data curation, investigation, formal analysis, supervision, funding acquisition, project administration, resources, writing – review and editing.

## Supporting information


S1.

S2.

## Data Availability

Data of the specimens processed at the University of Bristol (UK) are available at the University of Bristol data repository, data.bris, at https://doi.org/10.5523/bris.2kjrzkd739xgm25tm5m9sckpjw. The raw data of the specimen processed at Uppsala University (Sweden) are accessible at the ESRF Data Portal at https://doi.esrf.fr/10.15151/ESRF‐DC‐1992760470.
