# Supplementary material for: Investigating the Morphogenesis and Replacement of Lamprey Toothlets Using Synchrotron Imaging
Source: J Morphol. 2025 Oct 21;286(10):e70094. doi: 10.1002/jmor.70094 (PMC12541293; doi:10.1002/jmor.70094)
Supplement: Supplementary file 1 — S1. [file JMOR-286-e70094-s001.docx]

Supporting information

S1: Lamprey piston cartilage


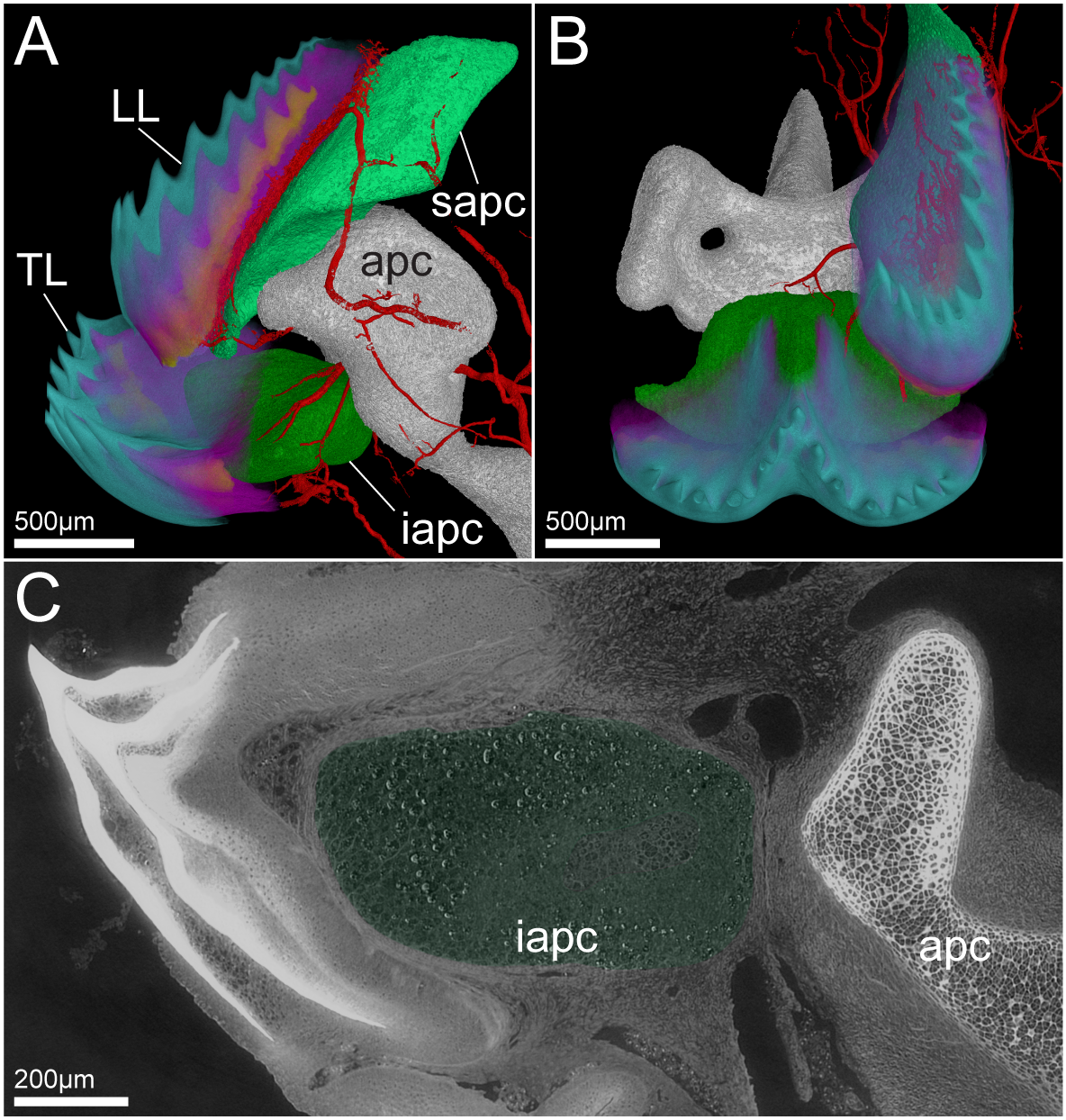


A, lateral and B, anterior rendering of the isolated tongue teeth, supporting cartilages, and vasculature (red) of a *P. marinus* juvenile (right LL and sapc not rendered). C, virtual thin section through the ventral tongue midline, contrasting the soft mucocartilage of the infraapical piston mucocartilage with the hard cartilage of the apical piston cartilage. apc = apical piston cartilage, iapc = infraapical piston mucocartilage, LL = longitudinal lingual lamina, sapc = supraapical piston cartilages, TL = transverse lingual lamina.
